# Species Classification for Neuroscience Literature Based on Span of Interest Using Sequence-to-Sequence Learning Model

**DOI:** 10.3389/fnhum.2020.00128

**Published:** 2020-04-21

**Authors:** Hongyin Zhu, Yi Zeng, Dongsheng Wang, Cunqing Huangfu

**Affiliations:** ^1^Research Center for Brain-Inspired Intelligence, Institute of Automation, Chinese Academy of Sciences, Beijing, China; ^2^School of Artificial Intelligence, University of Chinese Academy of Sciences, Beijing, China; ^3^Center for Excellence in Brain Science and Intelligence Technology Chinese Academy of Sciences, Shanghai, China; ^4^National Laboratory of Pattern Recognition, Institute of Automation, Chinese Academy of Science, Beijing, China; ^5^Department of Computer Science, University of Copenhagen, Copenhagen, Denmark

**Keywords:** brain science, neuroscience, cognitive computing, multi-label classification, corpus annotation, PubMed, linked brain data

## Abstract

Large-scale neuroscience literature call for effective methods to mine the knowledge from species perspective to link the brain and neuroscience communities, neurorobotics, computing devices, and AI research communities. Structured knowledge can motivate researchers to better understand the functionality and structure of the brain and link the related resources and components. However, the abstracts of massive scientific works do not explicitly mention the species. Therefore, in addition to dictionary-based methods, we need to mine species using cognitive computing models that are more like the human reading process, and these methods can take advantage of the rich information in the literature. We also enable the model to automatically distinguish whether the mentioned species is the main research subject. Distinguishing the two situations can generate value at different levels of knowledge management. We propose SpecExplorer project which is used to explore the knowledge associations of different species for brain and neuroscience. This project frees humans from the tedious task of classifying neuroscience literature by species. Species classification task belongs to the multi-label classification which is more complex than the single-label classification due to the correlation between labels. To resolve this problem, we present the sequence-to-sequence classification framework to adaptively assign multiple species to the literature. To model the structure information of documents, we propose the hierarchical attentive decoding (HAD) to extract span of interest (SOI) for predicting each species. We create three datasets from PubMed and PMC corpora. We present two versions of annotation criteria (mention-based annotation and semantic-based annotation) for species research. Experiments demonstrate that our approach achieves improvements in the final results. Finally, we perform species-based analysis of brain diseases, brain cognitive functions, and proteins related to the hippocampus and provide potential research directions for certain species.

## 1. Introduction

Managing neuroscience literature from species perspective is an innovative and important research task for understanding the functionality and structure of the brain. Species information in scientific works can be used to organize knowledge facts in the Linked Brain Data^1^ (LBD) (Zeng et al., 2014b) scheme, and then the system composed of brain and neuroscience communities (Ascoli et al., 2007; Gardner et al., 2008; Imam et al., 2012; Sunkin et al., 2012; Larson and Martone, 2013; Poo et al., 2016), neurorobotics, and other devices can automatically utilize species knowledge on the Internet by accessing the API provided by the LBD platform. For example, brain science knowledge of different species can be used to build brain simulation cloud computing platforms for different animals (Liu et al., 2016), monkey brain-inspired neurorobotics (Zeng et al., 2018), Drosophila brain-inspired Unmanned Aerial Vehicle (UAV) (Zhao et al., 2018), neuroimaging (Zeng et al., 2014a), and help neuroscientists design biological experiments (Poo et al., 2016). Internet of Things for brain science aims to link the brain-related data and devices to the Internet and help research and protect the brain. Our research opens up new opportunities for understanding and exploring the brain of different species to promote brain and neuroscience research. The species classification task is to assign pre-defined species labels to neuroscience literature that does not explicitly mention the species. This technology can be used to classify and organize neuroscience literature based on the species to help researchers and devices easily compare the similarities and differences between different species for linking the brain and neuroscience communities and different devices. The knowledge about certain species can also help find solutions to address some of the major health problems in humans, e.g., the HIV (Micci and Paiardini, 2016), the Jenner vaccine (Riedel, 2005), the Parkinson's disease (Bailey, 2006), etc.

The use of model organisms for human research purposes is commonplace—researchers can study these organisms in ways that are unethical or impractical in humans. Model organisms represent the species that have been extensively studied to understand specific biological phenomena and are usually easy to maintain and breed in a laboratory setting. In this paper, as an illustrative example, we focus on 23 types of representative animal models selected from Neuromorpho.org, i.e., “Agouti, Blowfly, Elegans, Cat, Chicken, Cricket, Dragonfly, *Drosophila melanogaster*, Elephant, Frog, Goldfish, Guinea pig, Human, Monkey, Moth, Mouse, Rabbit, Rat, Salamander, Sheep, Spiny lobster, Turtle, Zebrafish”. Many scientific works do not explicitly mention research species, which poses challenges for large-scale automated species extraction and analysis. Although some species can be inferred by manual reading and analysis of other information in the literature, such as target gene terms, organs, and functions, it is already difficult for humans to read a hundred articles. Analyzing millions of literature in this way is almost impossible. When classifying these documents, the human brain uses not only the brain's dictionary matching mechanism but also other mechanisms (such as attention and memory). The secondary challenge is how to guess various species at once. The research of other species is crucial for the study of brain and neuroscience. Faced with large-scale literature, it is inefficient to manually summarize species or to infer species using complex processes.

Species information is one of the most basic information that researchers are concerned about. (1) Researchers based on model organisms first focus on what species the research is based on. Because the species studied in the paper determine whether this paper has reference value or impact on their research. When research problems shift from frontier species to later species, a lot of species matching work is needed. It would be great if the species could be identified automatically. For example, specific genes related to working memory have been studied in *Drosophila melanogaster*, and they have also been found in mice, but no experiments have been performed. If the researcher doing the mouse experiment wants to search all the genes that have been studied in other species, or if he wants to search whether the specific genes present in mice have been studied in other species, then he first needs to know which species were studied in each article. Species are important information in biological research because each species has different characteristics, the research area suitable for each species is different, and the infrastructure investment (e.g., smart animal house, humidity and temperature control devices, laboratory instrument, etc.) of each species is also different. For example, zebrafish are suitable for exploring developmental problems, and fruit flies are more likely to perform genetically modified experiments. It is difficult to use mice to study developmental problems. It is important and instructive to make full use of species information for knowledge integration. (2) For researchers who do not consider too much species information, they also need to be aware of the importance of species in their research. If researchers want to write a review, such as a survey of mice or fruit flies, the need to use such a toolkit to eliminate many unnecessary papers. (3) If researchers want to build an automated literature analysis system in a certain field, the lack of species information will lead to confusion of knowledge on the Internet. In subsequent applications, users cannot get the results they are searching for. Machines simply cannot distinguish which species the knowledge belongs to, so this system cannot be easily accomplished.

Brain science knowledge urgently needs to be managed from a species perspective. Otherwise, this knowledge will be mixed, which will seriously affect subsequent applications and elements, including biologists/researchers who perform literature analysis and the automated literature analysis systems on the Internet. We need to use the knowledge of other species to solve the problems of humans. Categorizing several documents manually does not yield much valuable information. Categorizing large-scale literature by species will help harness the knowledge of other species to solve the problems of humans. This paper proposes a framework that can effectively process large-scale documents, improves the efficiency of literature analysis, and organizes the brain science knowledge based on species of interest. This framework uses not only species mentions and genetic terms but also cognitive computing models to process the contextual expressions and span of interest in the text. Our work has greatly improved the efficiency of species analysis and data transmission on the Internet.

This task can be formulated as two different task schemes, the text classification scheme (discriminative model) and the text summarization scheme (generative model). The text classification scheme classifies a document into different species, while the text summarization scheme summarizes the document from a species view and naturally considers the label correlation. The text classification scheme is easier because a document can be encoded as a fixed-length vector to retain the main information. The challenge is how to emphasize effective information about species in a long document. Note that this is a multi-label classification (MLC) task since a scientific work may be related to two or more species. The text summarization scheme is more like the human reading process because when humans read the paper, we gradually discover each species by mapping to different parts of the paper. Although the labels are obtained in a certain order, this order is not considered in evaluation—and this is not needed, as it is being used as a MLC problem. Inspired by the human reading process, the text summarization model gradually generates each species by attending to the span of interest (SOI) and considers the correlation between the tags. SOI in text is equivalent to region of interest (ROI) (Girshick et al., 2014; Girshick, 2015; Ren et al., 2015; He et al., 2017) in a picture. ROI is widely used in object detection of computer vision (CV) and it can be any particular portion of the image that seems important for the task. Here, we use SOI to represent the important text spans for species prediction.

The PubMed^2^ provides the citations of references and abstracts of biomedical literature from MEDLINE, life science journals, and online books. The PubMed Central (PMC)^3^ archives publicly accessible full-text articles of biomedical and life sciences journal literature. The research project of this paper is mainly about knowledge linking and extraction in the field of brain and neuroscience. Linked Brain Data (Zeng et al., 2014b, 2016; Zhu et al., 2016b,c) is an effort for extracting, integrating, linking and analyzing brain and neuroscience data and knowledge from multiple scale and multiple data sources. This platform focuses on the associations among brain regions, brain diseases, cognitive functions, neurons, proteins, and neurotransmitters. There are more than 2,339,898 relational triples in the LBD platform, such as (Hippocampus, relatedTo, Alzheimer's disease), (Hippocampus, relatedTo, Associative memory). These relations are machine-readable structured knowledge. This paper can organize massive structured brain science knowledge according to different species, thereby forming the structured species knowledge, which can be considered as 4-ary, e.g., (Hippocampus, relatedTo, Alzheimer's disease, Human) or (Hippocampus, relatedTo, Alzheimer's disease, Monkey). The proposed approach can facilitate the cross-species brain science research. The LBD platform provides services to connect the brain and neuroscience communities and devices.

A commonly used multi-label approach is the binary method (Fan and Lin, 2007) which builds a decision function for each class. Despite the success of the MLC scheme, it is often necessary to find a threshold to convert the probability value into a true/false flag for each class so that we can select a subset of the species as the final result. The thresholds for different species are usually different, and the final result is affected by the hard threshold. Finding globally optimal thresholds (Fan and Lin, 2007) for all classes is complicated. Inspired by Yang et al. (2018), we propose the sequence-to-sequence classification (SeqC) framework. Different from the MLC scheme, our SeqC framework does not need to search the thresholds because each step only outputs the most probable label by emphasizing SOIs. When there are no more species, this model will output the stop tag (Bahdanau et al., 2015). Abstractive summarization models usually have a ground truth sequence to learn how to paraphrase the main content of the passage and may use the teacher forcing (Williams and Zipser, 1989) and the scheduled sampling (Bengio et al., 2015) to improve the model performance. In contrast, this task only has class labels without the sequence order, so we convert species labels into virtual species sequences in a fixed order. During the model evaluation, we do not consider the label order.

MLC is more complex than single-label classification in that the labels tend to be correlated and different parts of a document have different contributions when predicting labels. Our decoder considers the correlations between species by processing species dependencies through LSTM units. A document can be very long, which poses a challenge for the one-level encoding model. Besides, not all sentences help to predict the species and not all words contribute equally to a sentence. To solve these two problems, we integrate the hierarchical document encoding and hierarchical attentive decoding (HAD) into the sequence-to-sequence model. We consider the word- and sentence/section- levels. Besides, simple MLC models only generate a vector representation that calculates an attention distribution over the document. Different species are usually associated with different parts of the document, so simple MLC models cannot adaptively attend to different parts of the document for different species, which potentially limits the performance. In contrast, our sequence-to-sequence classification model allows each species prediction to attend to different parts of the document.

To train and evaluate models, we label the PubMed and PMC corpora^4^. We present two versions of annotation criteria (mention-based annotation and semantic-based annotation). This paper is organized below. Section 3 describes the core modules of this framework. Section 4 describes the labeled datasets and experimental analysis. The major contributions of this paper can be summarized below.

- This paper formulates a new task, species classification in neuroscience literature. We propose the SeqC framework to classify neuroscience literature based on SOIs. This study improves the transfer efficiency of brain science knowledge on the Internet and opens up opportunities for brain science text mining from the species perspective.
- Our approach integrates the hierarchical document modeling and hierarchical attentive decoding to model the document structure and extract informative SOIs related to species. This framework supports both dictionary-based method and various deep learning models.
- We create three datasets which label 23 types of representative species in the PubMed and the PMC corpora. We propose two versions of annotation standards to facilitate the use of knowledge extraction in brain science text mining. This process is semi-automated and easily extendable to greater sets of species.

## 2. Related Work

Some works use the knowledge of different animals to resolve biological and biomedical questions. The species information can be used to manage the facts in a knowledge base to support the research of brain and neuroscience, such as the Brain Knowledge Engine^5^ (Zhu et al., 2016a). They organize the knowledge with species meta-data and explore the multi-scale nervous systems, cognitive functions and diseases of different species for linking brain and neuroscience communities, neurorobotics, brain simulation cloud computing platform, and other devices on the Internet by accessing the API. Norouzzadeh et al. (2018) propose a method to identify the location and behavior of animals from pictures to study and conserve ecosystems. McNaughton et al. (1983) study the contributions of position, direction, and velocity to single unit activity in the hippocampus of rats. Leach et al. (1996) found that blockade of the inhibitory effects of CTLA-4 can allow for, and potentiate, effective immune responses against tumor cells on mice. The above two contributions won the Nobel Prizes in Medicine because they have profound implications on human biomedical research. The animal information is also helpful for the study of the welfare of the animals, and the concept of animal rights (Andersen and Winter, 2017).

The technologies for the Internet of Things (Gochhayat et al., 2019; Kumar et al., 2019; Bebortta et al., 2020; Qian et al., 2020) are also widely used in different domains for understanding the functionality and structure of the brain and address some problems in human daily life. De Albuquerque et al. (2017) investigate the applications of brain computer interface systems. Some IoT frameworks are proposed to analyze the brain signals, such as brain CT images (Jaiswal et al., 2019; Sarmento et al., 2020; Vasconcelos et al., 2020), MRI (Mallick et al., 2019; Arunkumar et al., 2020), etc. Many applications benefit human daily life. Innovative algorithms for improving video streaming are proposed in the Internet of Multimedia Things (IoMT) and Internet of Health Things (IoHT) to optimize the Telemedicine and medical quality of service (m-QoS) (Sodhro et al., 2018). Sodhro et al. (2019a) propose the QGSRA algorithm to alleviate fluctuation in the wireless channel to support multimedia transmission. Using artificial intelligence algorithms to solve accurate resource management and energy efficiency issues (Sodhro et al., 2017, 2019b) is an important aspect of implementing the Internet of Things.

The NCBI Taxonomy^6^ (Federhen, 2011) is a curated classification and nomenclature for all of the organisms in the public sequence databases. It accounts for about 10% of the described species of life on the planet. It includes more than 234,991 species with formal names and another 405,546 species with informal names. Currently, the experiments of this paper focus on the 23 model organisms because there are systematic research methods for these species. Bada et al. (2012) create the Colorado Richly Annotated Full-Text (CRAFT) Corpus which contains 97 articles and annotates the concepts from 9 well-known biomedical ontologies and terminologies. Funk et al. (2014) evaluate dictionary-based concept recognizers on eight biomedical ontologies in the CRAFT dataset. Biomedical natural language processing (BioNLP) (Ananiadou and McNaught, 2006; Cohen and Demner-Fushman, 2014; Wei et al., 2015) aims to enable computers to efficiently read the vast amount of the literature and extract key knowledge about specific topics. There are some BioNLP tasks and corpora in the context of the BioCreative and BioNLP shared tasks. BioNLP (open) shared tasks (Dubitzky et al., 2013) contains a series of computational tasks of biomedical text mining (TM), evaluations, and workshops. Critical Assessment of Information Extraction in Biology (BioCreative) (Hirschman et al., 2005; Hemati and Mehler, 2019) includes assessments of biological domain information extraction and text mining development across the community.

BioNLP has achieved substantial progress on many tasks (Ananiadou and McNaught, 2006; Hunter and Cohen, 2006; Jensen et al., 2006), such as named entity recognition, information extraction, information retrieval, corpora annotation, evaluation, etc. These researches open up opportunities to integrate biomedical text mining with knowledge engineering and data mining. Many NLP techniques can be used to extract linguistic features from text in different languages for model learning, such as part-of-speech tagging, word segmentation, linguistic parsing (Manning et al., 2014; Zheng et al., 2016; Che et al., 2018; Li et al., 2019; Wang et al., 2020), etc. There are some researches on text mining in the genomics domain (Zweigenbaum et al., 2007), e.g., identifying gene/protein names and their relations. Hersh (2008) introduce the methods and challenges in many aspects of health and biomedical information retrieval systems. Bodenreider (2008) describe the role of biomedical ontologies in knowledge management, data integration, and decision support. There are some ontologies, such as SNOMED CT, the Logical Observation Identifiers, Names, and Codes (LOINC), the Foundational Model of Anatomy, the Gene Ontology, RxNorm, the National Cancer Institute Thesaurus, the International Classification of Diseases, the Medical Subject Headings (MeSH), and the Unified Medical Language System (UMLS). Smith et al. (2007) introduce the shared principles governing ontology development in the Open Biomedical Ontologies (OBO). Curtis et al. (2005), Khatri and Drăghici (2005), and Huang et al. (2008) use microarray technology and Gene Ontology (GO) terms to analyze the gene expression to characterize biological processes and identify the mechanisms that underlie diseases.

A commonly used multi-label approach is the binary method, which constructs a decision function for each class. Fan and Lin (2007) present a method to adjust the decision thresholds for each class. Zhang and Zhou (2007) propose the BP-MLL with a fully-connected neural network and a pairwise ranking loss function. Kim (2014) proposes the one layer CNN architecture with multiple filter width to encode both task-specific and static vectors. Nam et al. (2014) propose a neural network using cross-entropy loss instead of the ranking loss. Kurata et al. (2016) utilize word embeddings based on CNN to capture label correlations. Yang et al. (2016) propose a hierarchical attention network (HAN) to encode the sentence representation and document representation. They experimented with IMDB reviews, Amazon reviews, etc. for sentiment estimation and topic classification (Di Buccio et al., 2018; Tiwari and Melucci, 2018a,b, 2019a,b). Our model also considers the hierarchical attention, but the difference is that our model uses a decoder to resolve the multi-label classification problem and to calculate the hierarchical attention. Our proposed method uses the HAD mechanism in the decoder for each species prediction, while HAN calculates the attention in the encoding process. Besides, our model considers the discourse sections structure in scientific works during the decoding process. Liu et al. (2017) present a variant of CNN based approach to extreme multi-label text classification. Chen et al. (2017) propose a method to ensemble the CNN networks to capture diverse information on different nets. See et al. (2017) present the pointer generator network for text summarization. Yang et al. (2018) propose a sequence generation model for MLC. Cohan et al. (2018) propose a discourse-aware attention model for text summarization. They consider each section as a sequence and attending to the sequences of words. Inspired by the above studies, we integrate hierarchical document modeling, sequence-to-sequence model, and HAD into our species classification model.

## 3. Methods

First, we give an overview of the model. Second, we describe data acquisition, processing, and corpus annotation of the PubMed and PMC literature. Then, we explain in detail the SeqC framework of encoder and decoder which includes the sequence-to-sequence scheme and the hierarchical attentive decoding mechanism. Finally, we introduce the training method.

### 3.1. Overview

First, we define some notations and describe the species classification task. Given the predefined *m* species *L* = {*c*_1_, *c*_2_, …, *c*_*m*_} and a scientific work (neuroscience literature), our model assigns a subset of species to this document. More formally, each document has a list of predefined species candidates {*y*_1_, *y*_2_, …, *y*_*m*_}, where the label of the *i*-th species (*c*_*i*_) is *y*_*i*_ ∈ {0, 1} with 1 denotes a positive class and 0 otherwise. Our goal is to learn a model that can select the possible species subset involved in this scientific work. From the perspective of sequence-to-sequence model, this task can be modeled as finding an optimal species combination y^*^ that maximizes the conditional probability *p*(*y*|*x*), which is calculated as follows.

(1) $$\begin{array}{rc} p\left(y|x,\theta \right) & =\overset{m}{\underset{i=1}{\prod }}p\left(y_{i}|y_{1},y_{2},\ldots ,y_{i-1},x,\theta \right) \end{array}$$

where θ is the model parameter. The loss of the whole dataset can be calculated as Equation (2). We sort the label sequence of each sample according to the label frequency in the training set, with the higher frequency labels ranked front. For multi-label classification problems, the order of the labels is not needed for the result evaluation. We tested several methods to sort the labels and found that the results were almost the same.

(2) $$\begin{array}{rc} L\left(\theta \right) & =\underset{j}{\sum }p\left(y^{j}|x^{j},\theta \right) \end{array}$$

where *j* is the *j*-th document.

(3) $$\begin{array}{r} y^{*}=\arg\underset{y\in Y\left(z\right)}{\max}\log p\left(y|x,\theta \right) \end{array}$$

where *Y*(*z*) denotes 2^*m*^ possible combinations.

**Figure 1:**
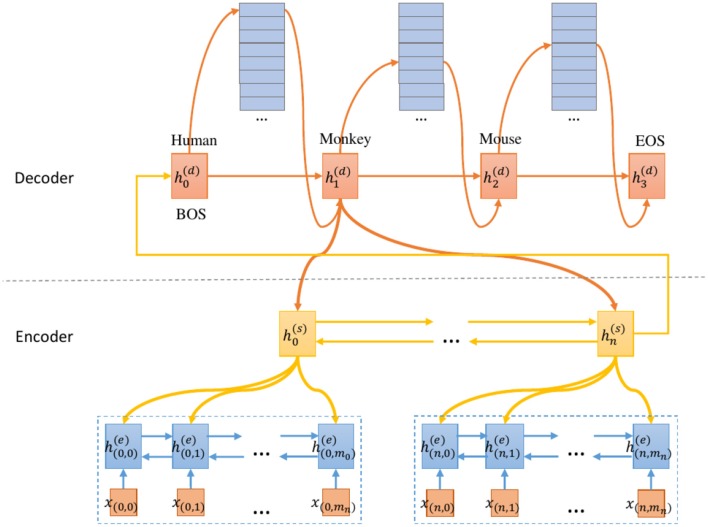
SeqC framework overview.

An overview of our proposed model is shown in Figure 1. Our main effort lies in designing a model that predicts each species by emphasizing SOI from the document. First, we convert the ground truth label into a species combination sequence. This allows the model to predict each species sequentially. Besides, the beginning symbol (BOS) and end symbol (EOS) are added to the head and tail of the species labels, respectively. Second, we use the two-level encoder to generate the contextual representation of the sentence/section and the document respectively. Finally, the decoder predicts each species by using the HAD mechanism.

This model can be seen as a simplified version of the neural abstractive text summarization model. Text summarization has a larger vocabulary for summarizing the main content, while the size of our vocabulary is 23. Text summarization allows the same words appear repeatedly in the output, while in our model each class label only appears once, so it reduces the repetition problem (See et al., 2017) in text summarization. Text summarization has the problem of out-of-vocabulary (OOV) words and uses the copy mechanism (See et al., 2017) to solve it, while our model does not have this problem since all the labels are fixed. In summary, this approach is promising in this task since this task is well-defined under the sequence-to-sequence classification scheme.

### 3.2. Data Processing

#### 3.2.1. Data Acquisition and Preprocessing

To obtain the neuroscience literature, we download all biomedical literature from 1987 to 2019 on the PubMed^7^ and PMC^8^. Then, we retrieve the biomedical literature related to neuroscience. We tokenize the documents and match the case-insensitive prefix (i.e., *brain,neuron,neural,neuro,cerebral*) at the word level.

In order to reduce the impact of the references and additional sections, we analyze the XML tag name and use the regular expression to extract the PMID/PMC, article title, abstract, keywords, article body, and date. We deleted tables to only preserve the textual content. We also convert XML escape characters into human-readable characters, for example, converting < to <, > to >, & to &, " to”, etc. Then we select the literature by matching the keywords in the title, abstract, and body of the article.

#### 3.2.2. Full-Category Sampling

We sample two sets of documents from PubMed and PMC corpora respectively. The set of articles in the PubMed corpus overlaps with the articles in the PMC corpus, given that the PMC articles would have a corresponding abstract in PubMed. To make the two datasets independent of each other, we removed the overlapping abstracts. The PubMed dataset contains 5,040/778/775 documents as the division of training/development/test (train/dev/test) sets. The PMC corpus contains 1,427/204/195 documents. In order to make the dataset cover all categories and better reflect the distribution of categories, we propose the full-category sampling (FCS) algorithm, as shown in Algorithm 1.

**Algorithm 1:** The full-category sampling algorithm

**Require:** The corpus *D* with species labels for each document, class support degree *s*, candidates number *n*
**Ensure:** The sampled dataset *samples*
1: Shuffle the corpus *D* and sample *n* candidates *D*′
2: *specDict* = {}, *samples* = []
3: **for** *i*←0, *D*′.*length*−1 **do**
4: *doc, tags* = *D*′[*i*]
5: *added* = False
6: **for** *j*←0, *tags*.*length*−1 **do**
7: **if** !*specDict*.contains(*tags*[*j*]) **then** *specDict*[*tags*[*j*]]=0
8: **end if**
9: **if** *specDict*[*tags*[*j*]] < *s* **then**
10: *specDict*[*tags*[*j*]]++
11: **if** *added* != True **then** Add *D*′[*i*] to *samples*
12: **end if**
13: *added*=True
14: **end if**
15: **end for**
16: **end for**

During the sampling process, we shuffle the documents and randomly select 50,000 documents as candidate documents. If the class support degree of species x (e.g., Mouse) reaches 400, this method no longer samples this species. The x denotes any pre-defined species. This class support degree denotes the maximum number of documents in each class. This method ensures that the dataset can cover all categories. The key insight of this algorithm is that it can prevent the oversampling of sparse classes.

We explain this algorithm. As shown in line 1, this method shuffles the corpus and randomly samples the candidate set. This operation prevents the oversampling of sparse classes. Otherwise, for sparse classes, this method will skip too many unrelated documents until enough samples of this class are obtained. Then, we initialize the *specDict* and *samples* to hold the sample results. Note that each sample is annotated with the mention-based annotation described in subsection 3.2.3. In lines 4–14, if the tag of the *i*-th document contains species x and the number of documents related to species x does not reach the class support degree *s*, the *i*-th document will be added to the dataset. Finally, *samples* contains the selected documents.

#### 3.2.3. Corpus Annotation

From the perspective of literary expression, the expression of related species is mainly divided into two types. First, some species are mentioned in the literature, such as monkeys, but monkeys themselves are not the main experimental subjects. Monkeys are associated with this study. This information can help find more comprehensive and instructive relevant knowledge. Second, this species is the main experimental subjects of the literature. This information can produce accurate semantic search results. Both cases have high research value. We create two versions of the dataset which are the mention-based annotation and the semantic-based annotation.

##### 3.2.3.1. Mention-based annotation

The first version (mention-based annotation) follows the criteria of species mention, which considers all the mentioned species as labels. More formally, let *c*_*i*_ ∈ *C* denote a predefined species, where *C* is the pre-specified species set. *s*_*j*_ ∈ *S* is a sample (i.e., an abstract or an article). If *s*_*j*_ mentions *c*_*i*_ (including one of its synonyms, variants, subspecies and its common alias from NCBI Taxonomy vocabulary), we assign *c*_*i*_ to *s*_*j*_. We consider the singular and plural forms of the species. We use the above dictionary-based method to label the entire dataset. Labeling documents that explicitly mention species is straightforward and efficient. The advantage is that it can find more relevant and comprehensive species to a study. After that, we can use these species labels as keys to efficiently retrieve the literature related to a specific species. This process avoids repeated computation and saves resources. The species tags of each article link massive documents. Users can utilize species tags to get more articles. This method is more complete and efficient than using words to retrieve plain text.

We also let three human annotators check the comprehensiveness and correctness of the species labeled for each sample. For example, some documents use other words related to humans, e.g., “humankind, humanity, humane, man, woman, men, women, male, female, patients." Overview articles also follow this annotation standard consistently, so they are considered relevant to the species mentioned. A conclusive dataset is generated using the combination of these annotations by an independent person.

The dictionary-based method may not perform well in the following situations. Sometimes, it is necessary to use context to determine whether “cricket” is a species or a game and whether “mouse” is an animal or a computer device. There are 18 PMC articles and 2 PubMed abstracts use “cricket” as the game. For example, “Hamstring injuries are not confined strictly to Australian Rules football but are also seen in soccer, athletics, hurling, **cricket** and touch football (Hoskins and Pollard, 2005).” There are 6 PMC articles and 1 PubMed abstract use “mouse” as the computer device. For example, “Total in-home computer use per day was calculated using **mouse** movement detection and averaged over a 1-month period surrounding the MRI (Silbert et al., 2016).” The weakness is that this standard may introduce some noisy species labels when they are not the main research subjects of the literature. This problem can be resolved by the following semantic-based annotation.

##### 3.2.3.2. Semantic-based annotation

The second version (semantic-based annotation) follows the criteria of expert knowledge. We let domain experts in the field of biology manually label the above PMC dataset based on the main research subjects of the article body. However, this process is costly and time-consuming, because annotators need to read the article and discuss the annotation standard. We add “cell,” “not applicable,” and “others” classes in that most cell-centric experiments share common methodologies. It is valuable to consider the “cell” as a class. For example, there are a lot of drug tests on cell or expression system related researches. Besides, a few papers did not study these species. We also need to use appropriate levels of species as the label to generate more valuable information. For the moth, considering a specific moth cannot generate much valuable information. The advantage of this standard is that articles retrieved using the primary research subject are more likely to contain satisfactory knowledge. However, the weakness is that the recall may not be high enough. For example, humans are not actually studied in some articles, but the research as a whole is done for the purpose of gaining insight into a disease that affects humans. There are 968 such documents without human labels. The mention-based annotation can make up for this problem. The mention-based annotation generally mine more species from these documents. Detailed standard is described in section 1 in the Supplementary Material^9^.

##### 3.2.3.3. Inferring species from the literature

To evaluate whether our model can infer species from the literature that does not mention species, we hid the species mentions and substituted them with the same symbol “*SPECIES*" to simulate the document that does not mention species. For example, masking “monkey" and “mouse” in a document (Cho et al., 2019), the sentence

We have established monkey NPC cell lines from induced pluripotent stem cells (iPSCs) that can differentiate into GABAergic neurons *in vitro* as well as in mouse brains without tumor formation.

becomes

We have established *SPECIES* NPC cell lines from induced pluripotent stem cells (iPSCs) that can differentiate into GABAergic neurons *in vitro* as well as in *SPECIES* brains without tumor formation.

Masked language models predict each masked token in the sentence, which is the token-level prediction. Different from the masked language model, we do not predict the masked token in the document, instead we predict each species only once and the prediction happens in the whole document, which is the document-level prediction. Masking species enables the model to learn how to use other information in the text to execute inference. Otherwise, the attention focuses on species words, not generating much valuable information. Besides, the performance of all models on the PubMed dataset is almost the same as using a dictionary-based method. In practice, this model does not need the above mask operation since we can input the original scientific work (with or without mentioning the species). To quantitatively analyze the inference performance, this way of data creation can reduce the risk of missing species. We also test our model when restoring the species mention. We keep the original files for human access. This would be critical for correct resolution.

### 3.3. Encoder

Our encoder extends the RNN encoder to the hierarchical RNN that captures the document structure. We first encode each sentence/section and then encode the document. The word-section level encoding is only used to model the article body. The abstract does not have section, but we unify these two modeling into one framework. Therefore, $h_{i}^{\left(s\right)}$ denotes sentence and section interchangeably. Formally, we encode the document as a vector based on the following equation:

(4) $$\begin{array}{r} h^{\left(doc\right)}=RNN_{doc}\left(h_{1}^{\left(s\right)},h_{2}^{\left(s\right)},\ldots ,h_{n}^{\left(s\right)}\right) \end{array}$$

*RNN*(·) represents a recurrent neural network whose final state is used to represent the input sequence. *n* is the number of sequences in the document. The superscript ^(*s*)^ and ^(*doc*)^ denote the sentence/section and the document representation respectively. $h_{i}^{\left(s\right)}$ is the representation of the *i*-th sequence, which is computed as follows.

(5) $$\begin{array}{r} h_{i}^{\left(s\right)}=RNN_{s}\left(x_{\left(i,1\right)},x_{\left(i,2\right)},\ldots ,x_{\left(i,m\right)}\right) \end{array}$$

where *x*_(*i, j*)_ is a word embedding of token *w*_(*i, j*)_ and *m* is the sequence length. The parameters of *RNN*_*s*_(·) are shared by all the sentences/sections. We use the single layer bidirectional LSTM for both *RNN*_*doc*_(·) and *RNN*_*s*_(·) to encode hidden states.

### 3.4. Decoder

#### 3.4.1. Sequence-to-Sequence Scheme

See et al. (2017) present the pointer-generator network for text summarization. Different from them, our decoder aims to model the correlation between species. At each step *t*, the decoder (a single-layer unidirectional LSTM) receives the species embedding of the previous step and the information of the input document. During training, the previous species comes from the ground truth label; at test time, the previous species is emitted by the decoder. The hidden state $h_{t}^{\left(d\right)}$ at time step *t* is computed as follows.

(6) $$\begin{array}{r} h_{t}^{\left(d\right)}=RNN_{dec}\left(\left[spec\left(y_{t-1}\right);c_{t-1}\right],h_{t-1}^{\left(d\right)}\right) \end{array}$$

where [;] denotes the concatenation operation. The superscript ^(*d*)^ denotes the decoder. *RNN*_*dec*_(·) is a uni-directional LSTM-RNN decoder. *spec*(*y*_*t*−1_) denotes the species embedding with the highest probability under the prediction distribution *y*_*t*−1_. *y*_*t*−1_ is the prediction of the previous step. *c*_*t*−1_ is the context vector generated from the input document using the hierarchical attention mechanism. *spec*(*y*_0_) is initialized to a trainable vector. *c*_0_ and $h_{0}^{\left(d\right)}$ are initialized to a zero vector and the document vector *h*^(*doc*)^ respectively.

#### 3.4.2. Hierarchical Attentive Decoding Mechanism

When the model predicts certain species, not all sentences/sections and words contribute equally. The attention mechanism can generate a context vector by attending to the SOIs of the document and aggregating their contextual representations. Modeling an article directly into a sequence of words cannot fully preserve the information and structure of the document. Discourse structure (Tang et al., 2015) information has proven effective in modeling document. Scientific works are usually composed of standard discourse sections structure describing the problem, methodology, experiments, conclusions, etc. Cohan et al. (2018) present a discourse-aware attention mechanism that generates better representation by incorporating discourse sections structure knowledge in the model architecture. We propose the HAD mechanism to consider discourse sections information for species prediction so that the model can extract important information from the literature more accurately based on the discourse sections, thus obtaining a better vector representation. Most literature only provides abstracts, so we use the HAD mechanism for the word and the sentence/section. When we process the full-text, our model uses the discourse sections structure, like (Cohan et al., 2018).

Specifically, the context vector related to the species information is computed as follows.

(7) $$\begin{array}{r} c_{t}=\overset{n}{\underset{i}{\sum }}\overset{m}{\underset{j}{\sum }}\alpha_{t\left(i,j\right)}h_{\left(i,j\right)}^{\left(e\right)} \end{array}$$

where $h_{\left(i,j\right)}^{\left(e\right)}$ is the hidden state of the encoder for the *j*-th word in the *i*-th section. The superscript ^(*e*)^ denotes the encoder. α_*t*(*i, j*)_ denotes the attention weight of the *j*-th word in the *i*-th section at the *t*-th step. The scalar weight α_*t*(*i, j*)_ is computed as follows.

(8) $$\begin{array}{r} \alpha_{t\left(i,j\right)}=\underset{\left(i,j\right)}{\text{softmax}}\left(\beta_{t\left(i\right)}\text{score}\left(h_{\left(i,j\right)}^{\left(e\right)},h_{t-1}^{\left(d\right)}\right)\right) \end{array}$$

where the score(·) function is the additive attention function, as shown in formula (10). β_*t*(*i*)_ is the weight of the *i*-th section at the *t*-th step. We parse the start and end positions of each section from the original literature files using the DOM parser so that we can find discourse sections.

(9) $$\begin{array}{r} \beta_{t\left(j\right)}=\underset{i}{\text{softmax}}\left(\text{score}\left(h_{i}^{\left(s\right)},h_{t-1}^{\left(d\right)}\right)\right) \end{array}$$

The correlation score is calculated by the additive attention (Bahdanau et al., 2015). $h_{i}^{\left(s\right)}$ denotes the hidden state of the *i*-th section.

(10) $$\begin{array}{r} \text{score}\left(h_{\left(i,j\right)}^{\left(e\right)},h_{t-1}^{\left(d\right)}\right)=v^{T}tanh\left(W_{1}h_{\left(i,j\right)}^{\left(e\right)}+W_{2}h_{t-1}^{\left(d\right)}+b^{\left(d\right)}\right) \end{array}$$

where *v* ∈ ℝ^τ^ is a weight vector. $W_{1},W_{2}\in {\mathbb{R}}^{\tau \times \tau }$ are weight matrices. *b*^(*d*)^ ∈ ℝ^τ^ is a bias vector.

### 3.5. Training Method

At the *t*-th decoding step, the vector $h_{t}^{\left(d\right)}$ generated by the decoder is used to predict the probability distribution of each class by the softmax function, as shown in Equation (11).

(11) $$\begin{array}{r} ŷ=\text{softmax}\left(Wh_{t}^{\left(d\right)}+b+I_{t}\right) \end{array}$$

where the *W* and *b* are the weight matrix and bias vector. $I_{t}\in {\mathbb{R}}^{m}$ is the mask vector that prevents the decoder from predicting repeated species.

(12) $$\begin{array}{r} {\left(I_{t}\right)}_{i}=\{\begin{array}{cc} -\infty , & \text{if species}y_{i}\;\text{has been predicted at previous time}\\ steps\\ 0, & \text{otherwise} \end{array} \end{array}$$

At the training time, the objective function is the cross-entropy loss as follows.

(13) $$\begin{array}{r} \underset{\Theta }{\min}L=-\overset{\left|D\right|}{\underset{i}{\sum }}\frac{1}{l^{\left(i\right)}}\overset{l^{\left(i\right)}}{\underset{t}{\sum }}y_{t}^{\left(i\right)}\cdot \log\left({ŷ}_{t}^{\left(i\right)}\right) \end{array}$$

where *i* is the document index and *t* is the decoder time step. Θ is the model parameter. |*D*| is the size of the training set. *l*^(*i*)^ is the decoder sequence length of *i*-th document. ${ŷ}_{t}^{\left(i\right)}$ is the predicted probability of ground truth class $y_{t}^{\left(i\right)}$ at the *t*-th time step. At test time, we use the beam search algorithm (Wiseman and Rush, 2016) to find the top-ranked prediction sequence.

## 4. Results

In this section, we conduct experiments on three datasets. We first introduce the datasets, evaluation metrics, implementation details. Then, we compare our method with baselines. Finally, we analyze the model components and experimental results.

### 4.1. Experimental Settings

#### 4.1.1. Dataset

##### 4.1.1.1. PubMed

**Figure 2:**
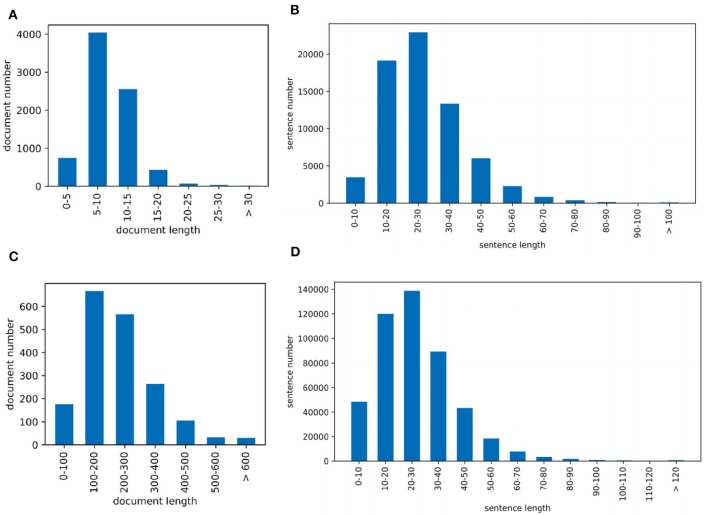
Dataset visualization where **(A)** is the PubMed sentence distribution of each document and **(B)** is the PubMed sentence length distribution and **(C)** is the PMC sentence distribution of each document and **(D)** is the PMC sentence length distribution.

**Figure 3:**
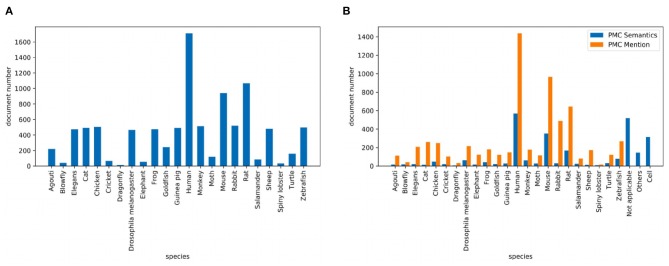
Species distribution where **(A)** is the species distribution of the PubMed dataset and **(B)** is the species distribution of the PMC dataset.

Corpus contains 2.55M abstracts, including 22.9M sentences, related to neuroscience science. 1.21M (47.5%) documents mention at least one pre-defined species using the mention-based annotation. The labels of these documents may not be complete, as the abstract may not mention all species. These documents can be used for further research in knowledge linking and extraction projects. We sample 5,040/778/775 documents as the experimental train/dev/test datasets. Figure 2A visualizes the distribution of sentence number of the abstract. The x and y axes are the sentence number in a scientific work and the count of scientific works that have the corresponding number of sentences respectively. Each document averagely contains 8.9 sentences. Figure 2B visualizes the sentence length distribution. Figure 3A visualizes the species distribution. “Human”, “Mouse,” and “Rat” are more frequent labels.

##### 4.1.1.2. PMC mention

Corpus consists of 0.43M articles, including 54.3M sentences, related to neuroscience science. 0.36M (83.5%) documents mention at least one pre-defined species. Annotating the entire corpus is costly and time-consuming, so we sample 1,427/204/195 documents as the train/dev/test datasets for our experiments. Figure 2C visualizes the distribution of sentence number of the paper. The sentence distribution varies over a wide range (14–3,087). Long documents occupy a small portion, so we merge the documents with more than 600 sentences. The criteria of this corpus is the species mention. Each document averagely contains 205.6 sentences. Figure 2D visualizes the sentence length distribution. Figure 3B visualizes the species distribution. “Human,” “Mouse,” “Rabbit,” and “Rat” are more frequent labels.

##### 4.1.1.3. PMC semantics

Dataset uses the same documents of the PMC Mention dataset. We let domain experts annotate these documents. The criteria of this version are based on expert knowledge. Figure 3B visualizes the species distribution. “Human,” “Mouse,” “Not applicable,” and “Cell” are more frequent labels.

#### 4.1.2. Evaluation

In single-label classification (1-of-n), the prediction can be either correct or wrong. Compared with the single-label classification, MLC is unique since the prediction can be partially correct (Venkatesan and Er, 2014). MLC requires different evaluation metrics to evaluate the partially correct. Following (Zhang and Zhou, 2007; Chen et al., 2017; Yang et al., 2018), we adopt the Hamming loss, micro-F1 score. Besides, we also measure the macro-F1 score and F1 per document. F1 per document would also be informative to measure document-level performance. This metric is calculated by averaging the precision, recall, and F1 of each document.

(14) $$\begin{array}{r} \text{Hamming}=\frac{1}{\left|N|\cdot |L\right|}\overset{\left|N\right|}{\underset{i=1}{\sum }}\overset{\left|L\right|}{\underset{j=1}{\sum }}\text{xor}\left(y_{\left(i,j\right)},t_{\left(i,j\right)}\right) \end{array}$$

##### 4.1.2.1. A. Hamming loss

Calculates the fraction of wrong labels. The lower the hamming loss, the better the performance is, as shown in formula (14). For an ideal classifier, the Hamming loss is 0.

(15) $$\begin{array}{r} \text{F1}=\frac{2\cdot \text{Precision}\cdot \text{Recall}}{\text{Precision}+\text{Recall}} \end{array}$$

##### 4.1.2.2. B. Micro-F1

Is the harmonic mean of micro-precision and micro-recall as formula (15). This metric calculates metrics globally by counting the total true positives, false negatives and false positives. This metric aggregates the contributions of all classes.

##### 4.1.2.3. C. Macro-F1

Computes the metric independently for each class and then take the average. This measurement treats all classes equally. We can evaluate the overall model performance for all classes.

#### 4.1.3. Implementation Details

Table 1 reports the main hyperparameters. We train the 200-D GloVe embedding on the whole PubMed and PMC corpora (3M documents). We did not update the pre-trained word embeddings during model training. For the character embeddings, we initialize each character as a 25-D vector. If using character Bi-LSTM, we set 50-D hidden state. If using character CNN, the convolution kernel width is 3, and we use max-pooling to generate 100-D vector representation. The Bi-LSTM dimension of encoder and decoder is 200-D. We use the Adam algorithm (Kingma and Ba, 2014) to train the model. The initial learning rate is 0.001. The size of species embedding is 200-D. We limit the sentence length to 128 and section length to 512 tokens. We conducted experiments on an Intel(R) Xeon(R) CPU E7-4830 v3 @ 2.10 GHz (Mem: 976G) and the GPU Tesla K40c (12G) and TITAN RTX (24G).

**Table 1:** The hyperparameter configuration.

**Hyperparameters**	**Value**
Character embedding	25
CNN kernel width	3
Encoder LSTM	100
Decoder LSTM	100
Dropout	0.5
Word embedding	GloVe.PubMed.200D
Epoch	100

### 4.2. Baseline Models

We compare our method with several baseline models. The Dictionary-based method uses string matching. To extract more species, the glossary of species includes species names, synonyms, variants, subspecies, and its common alias.

The LSTM (Zhang et al., 2015) and CNN (Kim, 2014) models consider the document as a sequence of words and generate a vector representation. The main difference is the components they choose to encode the document.

**Figure 4:**
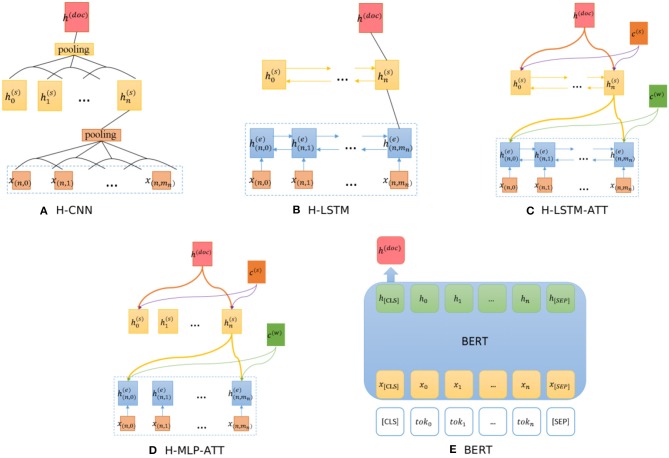
Architectures of baseline models. **(A)** H-CNN, **(B)** H-LSTM, **(C)** H-LSTM-ATT, **(D)** H-MLP-ATT, **(E)** BERT.

The hierarchical CNN (H-CNN) and hierarchical LSTM (H-LSTM) use word- and sentence- level encoders to model the document structure, as shown in Figures 4A,B respectively. This is a hierarchical version of CNN and LSTM models.

The H-LSTM-ATT, also known as the hierarchical attention network (HAN) (Yang et al., 2016), adds an attention mechanism to the H-LSTM to extract informative words, as shown in Figure 4C. *c*^(*w*)^ and *c*^(*s*)^ are the word- and sentence- level context vectors respectively, and they can be trained jointly. To evaluate the influence of the LSTM layer, the H-MLP-ATT replaces the LSTM layer with a single layer neural network with the ReLU activation function, as shown in Figure 4D. This network can be seen as the H-CNN-ATT with the kernel size of 1 × *d* where *d* is the vector dimension.

BERT (Devlin et al., 2019) is a pre-trained bidirectional transformer that has proven effective in various NLP tasks by fine-tuning the model. We use the representation of “[CLS]" to generate the document representation, as shown in Figure 4E. “[CLS]” stands for the representation of the class. Note that this model can only process up to 512 tokens.

### 4.3. Model Results

#### 4.3.1. Results of the PubMed Dataset

Table 2 lists the results on the PubMed dataset. A first observation is that hierarchical models (H-LSTM and H-CNN) achieve similar results with the corresponding single-level models (LSTM and CNN) on the PubMed dataset. CNN models achieve higher results than the LSTM models in document classification. H-LSTM-ATT achieves better results than H-LSTM. This means the attention mechanism is important on this task. H-LSTM-ATT outperforms H-MLP-ATT, which means the LSTM layer encodes more context information of the sentence and the document. H-LSTM-ATT outperforms CNN, which further proves the importance of attention mechanism.

**Table 2:** Results of species classification on the PubMed dataset.

**Algorithms**	**Hamming**	**Micro-F1**	**Macro-F1**
LSTM (Zhang et al., 2015)	0.0302	79.01	73.33
CNN (Kim, 2014)	0.0247	82.84	81.13
H-LSTM	0.0292	79.86	74.09
H-CNN	0.0245	82.87	79.04
H-MLP-ATT	0.0275	81.47	80.53
H-LSTM-ATT	0.0228	84.35	84.24
BERT	**0.0204**	**86.20**	**86.03**
**SeqC**	0.0247	83.57	82.42
Dictionary + Restore	0.0029	97.98	**99.50**
**SeqC + Restore**	**0.0007**	**99.46**	99.39

*Bold values represent the best results*.

**Figure 5:**
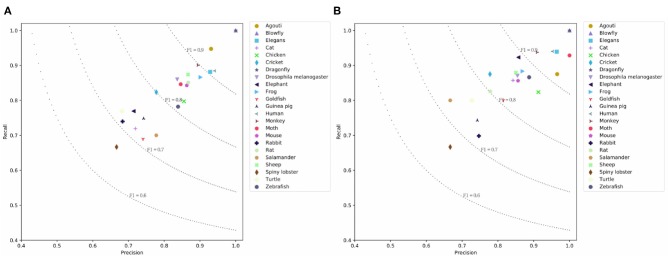
Prediction results on the PubMed dataset where **(A)** is the prediction results using SeqC and **(B)** is the prediction results using BERT.

BERT achieves the highest result because fine-tuning this model allows it to adapt to a new target task. BERT's P/R/F1 per document are 0.7843/0.7994/0.7847. The drawback is that the model cannot encode the document structure and has the highest computation costs. Our SeqC model achieves comparable results. The P/R/F1 per document are 0.7588/0.7774/0.7612. Figures 5A,B show the class-aware results of SeqC and BERT respectively. The x- and y-axes denote the precision and recall respectively. The dotted lines are the contours of the F1. We observe that BERT achieves higher results on “Elegans, Moth, Elephant, Cat, Goldfish” classes. SeqC achieves higher results on “Agouti, Rat” classes. Other species achieve comparable prediction results on both models.

The dictionary-based method is most computationally efficient and easier to use, but it can be difficult to accomplish this task without mentioning species in the document. We evaluate this method in the case of restoring (+ Restore) the mentions of species in the literature. The dictionary-based method is a good choice when directly extracting the mentions of species. Restoring the mentions also significantly improves the SeqC model results. This is because the model will pay attention to the mentions of species.

#### 4.3.2. Results of the PMC Mention Dataset

**Figure 6:**
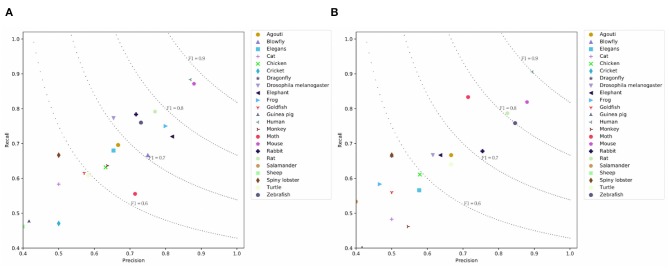
Prediction results on the PMC Mention dataset where **(A)** is the prediction results using SeqC + Discourse and **(B)** is the prediction results using BERT.

Table 3 presents the results on the PMC dataset. We observe that CNN and LSTM models achieve comparable results on the PMC dataset. BERT achieves similar micro-F1 score with the H-LSTM-ATT model, but the macro-F1 score is higher than other models. This means that the overall performance of BERT is more balanced across classes. The simple SeqC model cannot predict the masked species well. When the SeqC model considers the discourse sections structure (+ Discourse), this method outperforms all baselines. The discourse sections structure denotes the section-level structure in the article's body. This model uses the word-discourse HAD, that is, considering the word-section level attention. This means the section-level information is important for extracting the SOIs of the article. This is because certain sections (e.g., the experiments section) can find research species more effectively. Longer documents contain more noise, which poses challenges for model prediction. The P/R/F1 per document of SeqC + Discourse are 0.7598/0.6901/0.7021. As shown in Figures 6A,B, we observe that BERT achieves higher results on “Human, Moth, Zebrafish” classes. Our model achieves higher results on “Mouse, Frog, Elephant, Drosophila melanogaster, Blowfly, Elegans, Monkey, Goldfish, Cricket, Guinea pig” classes. Other species achieve comparable prediction results on both models.

**Table 3:** Results of species classification on the PMC Mention dataset.

**Algorithms**	**Hamming**	**Micro-F1**	**Macro-F1**
LSTM (Zhang et al., 2015)	0.0735	73.08	56.47
CNN (Kim, 2014)	0.0813	72.28	57.44
H-LSTM	0.0778	72.01	57.64
H-CNN	0.0760	72.78	57.05
H-MLP-ATT	0.0871	70.15	54.81
H-LSTM-ATT	0.0769	73.23	60.05
BERT	0.0767	73.93	63.02
**SeqC**	0.0889	70.26	55.91
**SeqC + Discourse**	**0.0655**	**76.81**	**64.41**
Dictionary + Restore	**0.0037**	**98.69**	**99.75**
**SeqC + Discourse + Restore**	0.0448	84.85	76.14

*Bold values represent the best results*.

When we restore the mentions of species in the literature, the dictionary-based method outperforms other methods. Restoring mentions of species also significantly improves the results of our model when we extract species from the article's body.

#### 4.3.3. Results of the PMC Semantics Dataset

**Figure 7:**
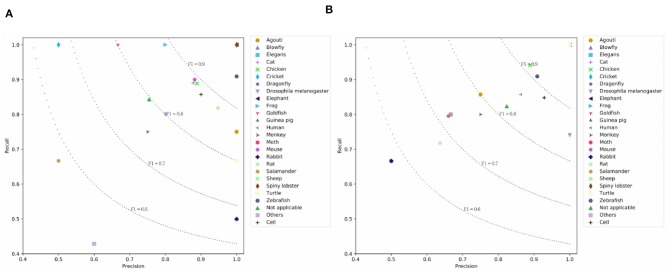
Prediction results on the PMC Semantics dataset where **(A)** is the prediction results using SeqC + Discourse and **(B)** is the prediction results using BERT.

Table 4 lists the results on the PMC Semantics dataset. We observe CNN models achieve higher results than the LSTM models. This means CNN units are good at capturing the internal semantics of documents. H-LSTM-ATT and H-CNN outperform the BERT. This means that the hierarchical modeling mechanism is good at capturing the document-level semantics. The simple SeqC does not perform well. The SeqC + Discourse achieves the highest performance. This means the section-level structure is more informative when modeling the article. This experiment proves our model is good at learning the semantic label of an article. As shown in Figures 7A,B, we observe that BERT achieves higher results on “Turtle, Salamander” classes. SeqC achieves higher results on “Spiny lobster, Zebrafish, Frog, Mouse, Rat, Goldfish, Cricket, Rabbit, Blowfly” classes. Other species achieve comparable prediction results on both models.

**Table 4:** Results of species classification on the PMC Semantics dataset.

**Algorithms**	**Hamming**	**Micro-F1**	**Macro-F1**
LSTM (Zhang et al., 2015)	0.0341	70.32	46.51
CNN (Kim, 2014)	0.0230	81.45	73.25
H-LSTM	0.0289	75.70	71.33
H-CNN	0.0213	82.17	72.05
H-MLP-ATT	0.0266	78.60	71.68
H-LSTM-ATT	0.0209	83.22	74.64
BERT	0.0230	81.57	72.12
**SeqC**	0.0246	80.91	70.34
**SeqC + Discourse**	**0.0203**	**84.03**	**74.75**
Dictionary + Restore	0.1270	42.50	35.38
**SeqC + Discourse + Restore**	**0.0189**	**85.41**	**79.03**

*Bold values represent the best results*.

The PMC mention dataset is easier because the criteria of species mention are straightforward. The PMC Semantics dataset is more difficult because the annotation criteria are more complicated. The SeqC model can be more flexible to focus on different words for each species, which is helpful to let the model learn the annotation rule. This model frees researchers from tedious work and automatically classifies the literature. This experiment further proves the effectiveness of our models. The P/R/F1 per document is 0.8102/0.8/0.8006.

### 4.4. Analysis and Discussion

#### 4.4.1. Ablation Study

To analyze the contributions and effects of different components, we perform ablation studies on the PubMed dataset, as shown in Table 5. The performance degrades by 1.83% micro-F1 without sentence-level attention (s-att). This is because the model cannot consider the sentence-level structure. The single-level attention only considers the word sequence, which assumes all sentences of a document are equally relevant for word selection. This setting limits the performance. When we remove the word-level attention (w-att), the performance drops by 2.02% micro-F1 and 4.28% macro-F1. This setting assumes that the contribution of all words in a sentence is the same, but the contribution of different sentences is different.

**Table 5:** The ablation results on the PubMed dataset.

**Model**	**Hamming**	**Micro-F1**	**Macro-F1**
SeqC	0.0247	83.57	82.42
–s-att	0.0274	81.74	82.06
–w-att	0.0274	81.55	78.14
–HAD (s-att,w-att)	0.0300	79.95	77.81
–HAD (s-att,w-att), decoder	0.0292	79.86	74.09

When we remove the HAD mechanism [s-att and word-level attention (w-att)], the performance drops by 3.62% micro-F1 and 4.61% macro-F1. This is because the model only uses the document vector to generate species and the decoder cannot attend to the document. When we remove the HAD mechanism and the decoder, the performance drops by 3.71% micro-F1 and 8.33% macro-F1. This is because the model becomes H-LSTM. The memory of a single document vector is limited.

#### 4.4.2. Results of Different Species

It is instructive to analyze the prediction result of different species. Figures 5A, 6A, 7A visualize the class-aware prediction results. The x- and y-axes represent the precision and recall respectively. The dotted lines denote the contours of the F1. For the PubMed dataset, we found “Dragonfly,” “Blowfly,” “Agouti,” “Elegans,” and “Human” are more easy to predict. The “Spiny lobster,” “Rabbit,” “Cat,” and “Goldfish” are more problematic. For the PMC Mention dataset, we observe the “Human” and “Mouse” are easier to extract. The “Sheep,” “Guinea pig,” “Cricket,” and “Cat” are more problematic. For the PMC Semantics dataset, we observe the “Elephant,” “Spiny lobster,” “Zebrafish” are easier to extract. The “Salamander” and “Others” are more problematic. We observe the prediction results are highly correlated to the class distribution.

As shown in Figure 3B, when we let experts annotate the corpus, the class imbalance problem has become more serious. This poses a challenge to the model. This phenomenon often occurs. Different versions of the annotated data have different class distributions. The forecasting of the results of the corpus annotation is important.

### 4.5. Species-Based Brain Cognitive Function, Brain Structure, and Protein Analysis

The hippocampus is a core brain region that is involved in many cognitive functions and brain diseases. The first part of Table 6 lists part of the data and knowledge about brain diseases of different species extracted and analyzed using the proposed method. These diseases are considered related to the hippocampal study. This knowledge is also freely accessible on the Internet. We observe that some brain diseases are related to hippocampus, such as “Alpers' disease,” “Anxiety,” “Autism,” “Brain edema,” “Cerebral artery occlusion,” “Lateral temporal epilepsy”, etc. The research about “Lateral temporal epilepsy” is mainly conducted on “Human,” “Rat,” “Mouse”, etc. Few studies are conducted based on the “Monkey,” “Guinea pig,” “Chicken,” etc. Experiments with some innovative species could be instructive for gaining innovative insights into this disease. We can trace back to the scientific works based on the “Guinea pig,” e.g., “The stimulation of 5-ht(1E) receptors and subsequent inhibition of adenylate cyclase activity in the DG suggests that 5-ht(1E) receptors may mediate regulation of hippocampal activity by 5-HT, making it a possible drug target for the treatment of neuropsychiatric disorders characterized by memory deficits (such as Alzheimer's disease) or as a target for the treatment of temporal lobe epilepsy (Klein and Teitler, 2012).”

**Table 6:** Some examples of brain diseases, brain cognitive functions and proteins related to the brain region “hippocampus” in different species, where the number behind the species is the number of related studies.

**Types**	**Examples**	**Species**
Braindiseases	Alpers' disease	Cat (2), Chicken (1), Human (46), Mouse (21), Rabbit (8), Rat (62), Sheep (2)
	Anxiety	Cat (9), Human (119), Monkey (8), Mouse (206), Rat (241)
	Autism	Human (11), Monkey (1), Mouse (22), Rat (16)
	Brain edema	Cat (1), Human (2), Mouse (11), Rabbit (4), Rat (33)
	Cerebral artery occlusion	Cat (1), Human (2), Mouse (22), Rat (38)
	Lateral temporal epilepsy	Cat (1), Chicken (2), Guinea pig (3), Human (348), Monkey (3), Mouse (102), Rat (253), Zebrafish (1)
Braincognitivefunctions	Associative learning	Human (19), Monkey (12), Mouse (24), Rabbit (5), Rat (34)
	Aversion	Cat (6), Human (52), Monkey (3), Mouse (96), Rabbit (6), Rat (356)
	Acuity	Human (1), Mouse (4), Rat (2)
	Concepts	Human(19), Monkey(2), Mouse(1), Rabbit(2), Rat(13)
	Decision making	Cat(1), Human(34), Mouse(6), Rat(26)
	Olfactory	Cat (5), Chicken (3), Frog (3), Guinea pig (7), Human (106), Monkey (7), Mouse (190), Rabbit (6), Rat (335), Sheep (8)
Proteins	Acetylcholine esterase	Cat (8), Guinea pig (8), Human (42), Monkey (6), Mouse (142), Rabbit (6), Rat (397)
	Adenosine deaminase	Human (1), Mouse (1), Rat (14)
	Adenylate cyclase	Cat (4), Chicken (1), Guinea pig (20), Human (18), Monkey (1), Mouse (31), Rabbit (1), Rat (150)
	Aromatase	Chicken (1), Human (15), Monkey (4), Mouse (30), Rat (42)
	Glutamine synthetase	Human (11), Mouse (10), Rabbit (2), Rat (41)
	Nitric oxide synthase	Guinea pig (9), Human (30), Mouse (89), Rat (240)

The second part of Table 6 lists part of the data and knowledge about cognitive functions of different species which are considered related to the hippocampal study. We observe that some cognitive functions are related to hippocampus, such as “Associative learning,” “Aversion,” “Acuity,” “Concepts,” “Decision making,” “Olfactory,” etc. Researchers prefer to conduct the researches for “Olfactory” on “Rat,” “Mouse,” “Human,” etc. Few studies are conducted based on the “Monkey,” “Sheep,” “Guinea pig”, etc. We found that research on monkeys' olfactory of smell may be relatively innovative. We can trace back to the scientific works based on the “Monkey,” e.g., “Early developmental events involving the olfactory and limbic system start and conclude possibly slightly early in primates than rodents, and we find a comparable early conclusion of primate hippocampal neurogenesis (as assessed by the relative number of Ki67 cells) suggesting a plateau to low levels at approximately 2 years of age in humans (Charvet and Finlay, 2018).”

It can be found in the third part of Table 6 that some proteins, such as “Acetylcholine esterase,” “Adenosine deaminase,” “Adenylate cyclase,” “Aromatase,” “Glutamine synthetase,” “Nitric oxide synthase,” etc., are related to the hippocampus. Researchers prefer to conduct the researches for “Nitric oxide synthase” on “Rat,” “Mouse,” “Human,” etc. Few studies are conducted based on the “Guinea pig.” We found that research on Guinea pig may be more instructive. For example, “Decreased nitric oxide synthase (NOS)-catalyzed formation of NO from L-arginine may be involved in ethanol teratogenesis involving the hippocampus (Gibson et al., 2000).”

### 4.6. Case Study

**Figure 8:**
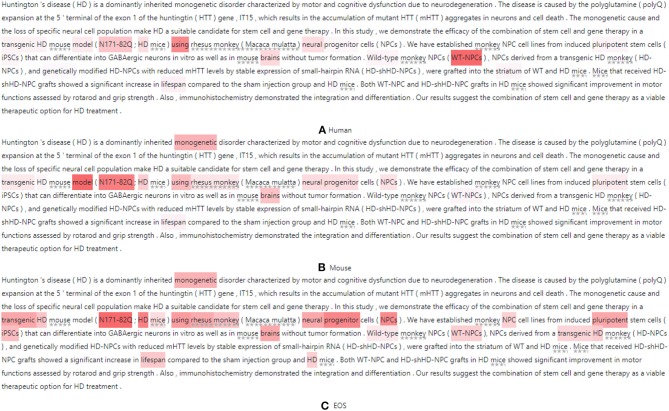
Visualization of SOIs when the model predicts **(A)** Human **(B)** Mouse and **(C)** EOS where redness indicates attention and the stars below the text indicate the masked species.

**Figure 9:**
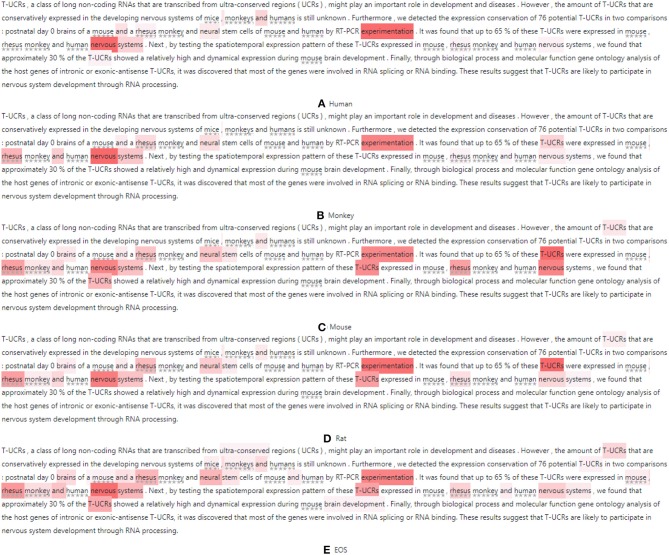
Visualization of SOIs when the model predicts **(A)** Human **(B)** Monkey **(C)** Mouse **(D)** Rat and **(E)** EOS where redness indicates attention and the stars below the text indicate the masked species.

It is instructive to analyze how the attention mechanism extracts SOIs to predict species. We choose two abstracts (Zhou et al., 2017; Cho et al., 2019) to visualize the attention distribution, as shown in Figures 8, 9. When the model predicts different species, it attends to different parts of the document. We restore the species names in the figure to better understand the samples. These species are marked with underlined stars.

For the first sample, this model first predicts “Human” by using the document representation. We observe this class is not mentioned in the abstract but is mentioned in the text so the “Human” can be assigned to this paper. This means our model can help infer more complete species. Some terms are potential topics in human-related research, e.g., “Huntington's disease,” “Cognitive dysfunction,” “huntingtin gene,” “monogenetic disorder,” etc. Figure 8A visualizes the attention distribution when predicting “Human.” The attention distribution (“transgenic HD, N171-82Q, HD, neural, WT-NPCs, iPSCs”) also contains information about the next species to be predicted, as this decoder sequentially models the correlation between species. When predicting “Mouse”, the attention weight of “monogenetic, N171-82Q, neural progenitor, NPCs, pluripotent” increases and the weight of “iPSCs, WT-NPCs” decreases, as shown in Figure 8B. When predicting “EOS,” token weights are distributed over all emphasized words and are most distracting, as shown in Figure 8C. This shows that the model attends to different words when predicting different species. The model also considers the correlation between labels and retains historical memory. However, this model misses “Monkey.”

For the second sample, when predicting “Human,” the model uses the document representation and attends to “neural, experimentation, nervous system, T-UCRs.” When predicting “Monkey,” the attention weights of “T-UCRs” and masked species words (“rhesus monkey”) are increased. When predicting “Mouse,” the weights of “T-UCRs, nervous systems, neural stem” are increased. When predicting “Rat,” the weights of “nervous systems, neural stem” are decreased. When predicting “EOS,” token weights are most distracting.

## 5. Conclusion

We propose the SeqC framework to classify neuroscience literature for linking brain and neuroscience communities and devices on the Internet. This study facilitates knowledge transfer and real-time data analysis over the Internet. The advantages are that it is possible to visualize words that are receiving attention to make the model interpretable. Additionally, this could be used to infer more complete names of species. We use hierarchical encoders to model the document structure. We use a decoder with the HAD mechanism to extract SOIs for different species. To evaluate model performance, we create three datasets for species research of brain and neuroscience. We resolve the problem of species annotation and present two versions of annotation criteria (mention-based annotation and semantic-based annotation). Limitations are that labels should be provided before, and that a manual tagging is needed. However, the process is semi-automated and can be easily extended to a wider variety of species.

This paper uses deep learning models to resolve the problem of species classification for neuroscience literature. The proposed cognitive computing model resolves this problem primarily by attending to the SOIs of a document. This approach can help predict species in the neuroscience literature. Structured species knowledge can be used to inspire researchers to better understand the knowledge associations in brain and neuroscience. In the future, the limitations of manual labeling can be alleviated by adding terms to the dictionary and using automatic model annotation. It seems promising to apply named entity recognition Zhu et al. (2019) models and attention mechanism to find more species names in the literature and perform open species extraction.

## Data Availability Statement

The datasets and codes generated for this study can be found in the Github https://github.com/sssgrowth/SPECIESEXPLORER.

## Author Contributions

YZ proposed the scientific question. HZ formalized the task, proposed the approach, annotated datasets and conducted the experiments. YZ contributed the domain terms, summarized the species, designed the ontology and upgraded the key insights of the model. HZ and YZ wrote the paper. DW collaborated to develop the models and data processing modules and annotate datasets. CH contributed the data annotation, upgraded the species annotation standard and discovered problems in the biological research process.

## Conflict of Interest

The authors declare that the research was conducted in the absence of any commercial or financial relationships that could be construed as a potential conflict of interest.

## Footnotes

^1^http://www.linked-brain-data.org

^2^https://www.ncbi.nlm.nih.gov/pubmed/

^3^https://www.ncbi.nlm.nih.gov/pmc/

^4^https://github.com/sssgrowth/SPECIESEXPLORER

^5^http://www.brain-knowledge-engine.org

^6^https://www.ncbi.nlm.nih.gov/taxonomy

^7^https://ftp.ncbi.nlm.nih.gov/pubmed/

^8^https://ftp.ncbi.nlm.nih.gov/pub/pmc/

^9^https://github.com/sssgrowth/SPECIESEXPLORER/blob/master/icon/appendix.pdf
