# Supplementary material for: Species Classification for Neuroscience Literature Based on Span of Interest Using Sequence-to-Sequence Learning Model
Source: Front Hum Neurosci. 2020 Apr 21;14:128. doi: 10.3389/fnhum.2020.00128 (PMC7187631; doi:10.3389/fnhum.2020.00128)
Supplement: Supplementary file 1 [file Data_Sheet_1.PDF]

Supplementary Material

1 STANDARD OF THE SEMANTIC-BASED ANNOTATION

Most labels denote single species, e.g., rat, mouse, C. elegans and D. Melanogaster, etc. We also need to use different levels of species as the label. For example, moth contains many types of species. A specific moth has been studied in only a few articles, so considering specific moth cannot find valuable related studies. Studies on different moths have many common features in the paper. Treating all the moths as one label can help summarize the research of moth while distinguishing all species will be impossible and will not generate more valuable analysis.

Most classes (species) are organisms, but we add a special class “cell”. As long as the article involves experiments on living cells, the article is assigned to “cell”. The reason for adding the “cell” class is that most cell-centric experiments use common methodologies, while the organisms that provide these cells are relatively unimportant. As long as the origin of the cell can be found in the article, the document is also assigned to the species of the original organism. Note that sometimes the original organism of cell lines cannot be directly extracted from the article. For example, in some researches, the article merely mentions that the experiment is carried out in HEK293 cell line. If we google HEK293, we could find that it is a human embryo kidney cell line from the human. Although we cannot read it from the article alone, this article will still be assigned to “cell” and “human”.

Organisms not included in the pre-defined species are labeled as “others”. If an article cannot be assigned to any of the species including “cell” and “others”, then the article will be labeled as “not applicable”. In this case, it is not appropriate to assert that an article is related to a particular creature. For example, an article develops research method of how to measure the glucose concentration of a solution, or an article generates a phylogenetic tree of all organisms in an order, or an article reports all species found in a certain ecosystem.

We determine whether an organism is associated with an article based on the following criteria:

(1) If an in vivo experiment is carried out on an organism, the article is assigned to the organism. If a living organism is used in the experiment as a tool, the article is not assigned to the organism. For example, in [Mori et al. (2012)], salamander is used as stimulation to induce an effect on the frog. This article will be only assigned to “frog” and not to “salamander”.

(2) Review articles are not assigned to any organism. First, the description of organisms in reviews also exists in research articles, while they do not indicate that the research articles are based on the organism. For example, if a research article is investigating the function of a gene in Drosophila Melanogaster, it would probably mention the research of the gene in mammals [Erdogan et al. (2016)]. However, this article is not based on mammals. Reviews would make a similar description about the gene. If an article is considered to be mammal-based, then the same description will indicate that the article of investigating Drosophila gene is based on the mammal. This is unreasonable. Some reviews discuss some organisms extensively [Banerjee et al. (2019)], and it seems reasonable to say that the review is based on a certain organism. However, the distinction between reviews that discuss certain organisms [Bell et al. (2012)] and reviews that discuss a phenomenon [Bell et al. (2016)] related to multiple organisms can be very vague.

Second, sometimes, in a review paper, it is impossible to tell which kind of organism the description is based on. Reviews sometimes do not mention which organism is related to the research. For example, in

this review [Sudhakaran and Ramaswami (2017)], many descriptions do not contain specific organism names. However, in research articles, researches based on certain organisms have specific methodologies and keywords. Analyzing the review paper may not identify the organism. However, analyzing the articles cited in the review paper can easily tell us which organism is related to the review, so excluding review from our research would not diminish the significance.

(3) If experiments in the article are based on cells, body fluid, or other body parts related to a certain organism, then the article is considered to be based on the organism. If a part of the organism is used as a tool rather than a research target, this article is not considered to be based on the organism, e.g., using antibody from rabbit, sheep, cattle, rat and hamster, or using serum from cattle, or using cornmeal and wheat in food.

(4) If the protein is used in the research and the protein is indeed the research target, then the research is considered to be based on the species of the protein. If the protein is expressed in another organism or cell, then the paper is usually considered to be based on the target organism or the organism of the cell. Because in this case, the protein has complicated interaction with the organism or cell. For most researches on this topic, observing the effect of the expression is a very important part of the research. Thus, the expression organism or cell cannot be merely considered as a tool. But if the expression organism or cell is just used to produce the protein for further experiments, like crystallization or enzyme catalysis, then they are not considered to be related to the article. If the experiment involves small molecule products like sugar, cellulose or other substrates that are not specifically produced by a species, then the article is not assigned to the organism.

(5) For researches on experimental methods, as long as the articles involve a demonstration or test of experimental methods on organisms, the study is considered to be based on the organism.

(6) For bioinformatics or systematic research, if the research focuses on a few organisms, e.g., comparing features between mouse, rat and human, and discussing them intensively, then the article is labeled with all these organisms. If the research draws conclusion on certain organism, then the article is labeled with the organism. If the research involves a lot of organisms and does not focus on a few of them, then the article is not labeled with the organism. For example, a research finds a lot of species through systematic searching a screening or collects all species in an orderly manner and forms a phylogenetic tree.

(7) Whether an article can be assigned to an organism still has gray area. For example, a research evaluates a protein produced by different kinds of cells belonging to different organisms, or a research uses DNA sequences from different organisms to deduce the function of a gene. Although the specific range of organism in these researches is uncertain, the academic tradition and methodology of these researches are explicit. In these cases, we insist that organisms need to best describe the academic tradition and methodology of the researches. We will use the same principles to label articles that are similar in academic traditions and methodologies.

REFERENCES

Banerjee, U., Girard, J. R., Goins, L. M., and Spratford, C. M. (2019). Drosophila as a genetic model for hematopoiesis. *Genetics* 211, 367–417

Bell, R. L., Hauser, S., Rodd, Z. A., Liang, T., Sari, Y., McClintick, J., et al. (2016). A genetic animal model of alcoholism for screening medications to treat addiction. In *International review of neurobiology* (Elsevier), vol. 126. 179–261

Bell, R. L., Sable, H. J., Colombo, G., Hyytia, P., Rodd, Z. A., and Lumeng, L. (2012). Animal models for medications development targeting alcohol abuse using selectively bred rat lines: neurobiological and pharmacological validity. *Pharmacology Biochemistry and Behavior* 103, 119–155

Erdogan, C. S., Hansen, B. W., and Vang, O. (2016). Are invertebrates relevant models in ageing research? focus on the effects of rapamycin on tor. *Mechanisms of ageing and development* 153, 22–29

Mori, T., Kitani, Y., Ogihara, J., Sugiyama, M., Yamamoto, G., Kishida, O., et al. (2012). Histological and ms spectrometric analyses of the modified tissue of bulgy form tadpoles induced by salamander predation. *Biology open* 1, 308–317

Sudhakaran, I. P. and Ramaswami, M. (2017). Long-term memory consolidation: The role of rna-binding proteins with prion-like domains. *RNA biology* 14, 568–586
